# The change of MRI indexes of brain glymphatic function and sleep status before and after repeated transcranial magnetic stimulation in insomnia disorder patients

**DOI:** 10.3389/fnins.2025.1545885

**Published:** 2025-05-30

**Authors:** Duo Gao, Zhengnan Zhang, Pingyong Feng, Lixia Zhou, Zuojun Geng, Caiying Li, Yifei Zhu, Haiqing Yang

**Affiliations:** ^1^Department of Medical Imaging, The Second Hospital of Hebei Medical University, Shijiazhuang, China; ^2^Hebei Key Laboratory of Medical Imaging, Shijiazhuang, China; ^3^Hebei Key Laboratory of Neurodegenerative Disease Mechanism, Shijiazhuang, China; ^4^Department of Psychosomatic Medicine, The Second Hospital of Hebei Medical University, Shijiazhuang, China

**Keywords:** diffusion-tensor imaging, insomnia disorder, brain glymphatic function, repetitive transcranial magnetic stimulation, sleep status

## Abstract

**Introduction:**

We aim to explore the associations between brain glymphatic function and sleep status in insomnia disorder patients (IDs), and to investigate whether repeated transcranial magnetic stimulation (rTMS) can improve sleep and glymphatic function.

**Methods:**

In this prospective study, consecutive IDs were enrolled and randomly classified into true rTMS group and sham rTMS group. Age- and sex-matched healthy control participants (HCs) were enrolled and examined between January 2023 and December 2023. Neuropsychological test included the Beck Depression Inventory (BDI), Beck Anxiety Inventory (BAI), the Montreal Cognitive Assessment (MOCA), and the Minimum Mental State Examination (MMSE). Sleep status was accessed using questionnaires and polysomnography (PSG) in all participants. Diffusion tensor imaging analysis along the perivascular space (DTI-ALPS) index of MRI was used to evaluate brain glymphatic function.

**Results:**

28 ID patients of true rTMS group, 9 ID patients of sham rTMS group, and 20 control participants were included. Before rTMS, both of the true/sham rTMS groups had lower DTI-ALPS than HCs. Multivariate linear regression models indicated that N2 sleep duration and the arousal index were independently associated with DTI-ALPS in all IDs. After rTMS, compared with the sham rTMS group, the sleep questionnaires, total sleep time, N2 sleep duration, the arousal index of PSG, and DTI-ALPS reflected that the treatment improved the sleep status and glymphatic function of IDs.

**Conclusion:**

Our research implies that N2 sleep duration and the arousal index were independently associated with lymphatic function. The rTMS therapy can improve glymphatic function and sleep status in IDs.

## Introduction

Insomnia disorder (ID), defined by persistent difficulty initiating or maintaining sleep (≥3 nights/week for ≥3 months) per to meet the international classification of sleep disorders-third edition (ICSD-3) diagnostic criteria (Sateia, 2014), is rising in prevalence and elevates cognitive impairment risk, particularly in middle-aged and elderly populations (Paul and Salas, 2024; Naha et al., 2024). Previous epidemiological studies have found that the prevalence of ID ranges from 30 to 35% of adults which may increase up to 75% in elderly (Nguyen et al., 2019; San and Arranz, 2024). ID is associated with a variety of neurocognitive deficits and psychosocial alterations.

Studies show sleep enhances glymphatic function, while insomnia disrupts it, compromising metabolic waste clearance (Mestre et al., 2020; Xie et al., 2013; Olsson et al., 2018; Krueger et al., 2016). Unlike previous studies of intravenous gadolinium-based contrast agents for lymphatic drainage (Zhou et al., 2020; Mortensen et al., 2019), magnetic resonance imaging (MRI)-based diffusion tensor imaging analysis along the perivascular space (DTI-ALPS) index provides an opportunity for non-invasive indirect evaluation of the glymphatic activity (Taoka et al., 2017b; Andica et al., 2023; Kamagata et al., 2022). The DTI-ALPS index, a diffusion tensor imaging metric quantifying perivascular space diffusivity relative to perpendicular fiber tract diffusivity (Taoka et al., 2017a), has emerged as a validated imaging biomarker for assessing brain glymphatic function (Ma et al., 2024; Siow et al., 2022; Xiong et al., 2024; Jin et al., 2024; Bae et al., 2023; Lin et al., 2024).

As a non-invasive neuro-modulatory technique, repetitive transcranial magnetic stimulation (rTMS) has been applied in sleep medicine, such as chronic insomnia, obstructive sleep apnea syndrome, restless leg syndrome and sleep deprivation, and it is considered safe and feasible (Walker and Stickgold, 2004). Currently, several studies have shown that rTMS is effective for ID (Qi et al., 2022; Zhang et al., 2022; Li et al., 2022; Shi et al., 2021; Wu et al., 2021; Zhang et al., 2018). Nevertheless, objective outcome measurements have not been deeply explored. The effectiveness findings based solely on polysomnography (PSG) lack consistency and may need to be combined with neuroimaging.

Therefore, the aims of this study were to (1) explore changes in cerebral lymphatic clearance function in ID patients and its association with sleep metrics of PSG. (2) the therapeutic effect was explored by comparing changes in sleep status and DTI-ALPS index in the ID patients before and after real and sham rTMS treatment.

## Methods

### Participants and subgroups

This single-center prospective study was approved by the institutional review board of the Second Hospital of Hebei Medical University. From January 2023 to December 2023, we prospectively recruited consecutive ID patients (IDs) from the psychosomatic medicine department. Age-and sex-matched healthy controls (HCs) were also recruited through advertisements from the physical examination department during the study period. All participants have signed the informed consents. The study procedures conformed to the Declaration of Helsinki.

The inclusion criteria of IDs were as follows: (1) aged from 18 to 65 years; (2) right-handed; (3) meet the criteria for IDs (Diagnostic and Statistical Manual of Mental Disorders, Fifth Edition, DSM-5) (First, 2013) (4) total score of Pittsburgh Sleep Quality Index (PSQI) > 5; (5) no hypnotic and psychoactive medications were taken at least 2 weeks before and during the study. The exclusion criteria were as follows: (1) patients with epilepsy, pregnant or lactating women; (2) accompanied by brain lesions or serious mental health disorder; (3) contraindications for 3.0 T MRI, such as claustrophobia, metal implants, etc. (4) unable to complete the forms. The inclusion criteria of HCs were as follows: (1) matched for age and gender; (2) no history of neurological or psychiatric diseases; (3) no taking any medications affecting the central nervous system; (4) the sleep quality rating scales were within normal range. Additional exclusion criteria for HCs were MRI examination showed obvious positive lesions and multiple abnormal signals.

All IDs were classified into true rTMS group and sham rTMS group. Finally, the study population was divided into the following three subgroups: HCs group, true rTMS group and sham rTMS group. Baseline of cognitive performance, sleep questionnaires, and PSG data and MRI examination were performed in all three groups. In addition, the true/sham rTMS groups received true/sham stimulation therapy, respectively. Cognitive performance, sleep questionnaires, MR images and PSG data were obtained within 3 days after treatment.

**Figure 1:**
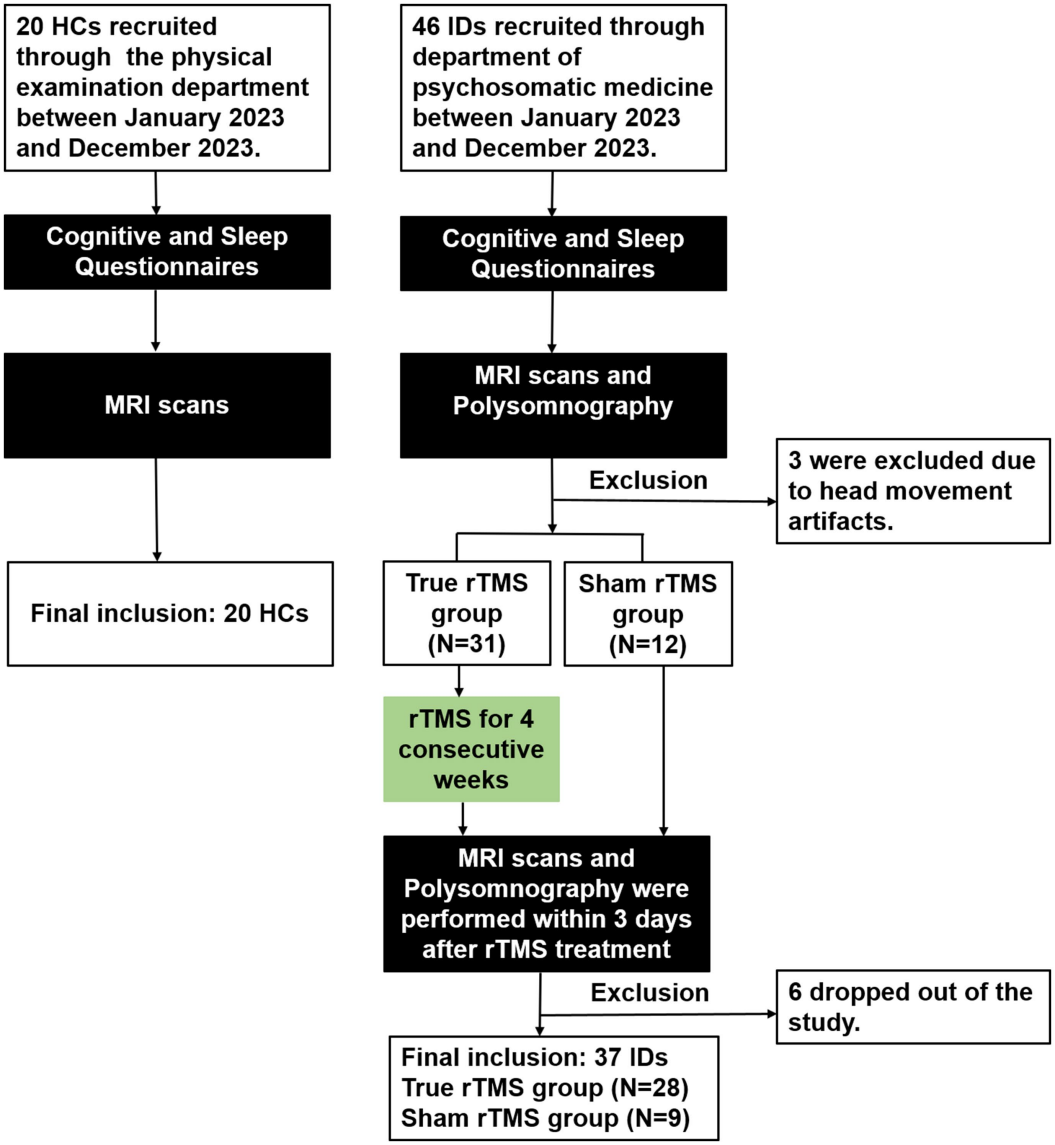
Flow diagram shows the participant selection process for healthy controls (HCs) (left) and insomnia disorder patients (IDs) (right).

Of the 46 IDs, 3 were ruled out because of head movement artifacts of MR images. Another 6 IDs were excluded due to early withdrawal from the study. True rTMS group (*n* = 28), sham rTMS group (*n* = 9) and HCs (*n* = 20) were eventually included in this study (flow diagram in Figure 1).

### Demographic data, cognitive performance and sleep questionnaires

All participants’ age, gender and education were recorded. Cognitive performance was measured by the Beck Depression Inventory (BDI), Beck Anxiety Inventory (BAI), Montreal Cognitive Assessment (MOCA) and Minimum Mental State Examination (MMSE). Three standardized self-rated sleep questionnaires were administered to all participants: (1) the Pittsburgh Sleep Quality Index (PSQI); (2) the Epworth Sleepiness Scale (ESS); (3) Insomnia Severity Index (ISI).

### rTMS

In this study, the MagPro R30 TMS stimulator with figure-of-eight coil (MagVenture, Denmark) was used for treatment. Active stimulation targeting the left dorsolateral prefrontal cortex (Li et al., 2022; Qi et al., 2022; Zhang et al., 2022; Lanza et al., 2023) was performed using the “5 cm rule” for localization. The TMS parameters were set at the frequency of 1 Hz and the stimulation intensity was 80% of the resting movement threshold. The stimulus consists of 10 pulses per string, with an interval of 2 s, applying a total of 150 strings, and producing a total of 1,500 stimulation pulses within 30 min. Treatment was performed once a day, 5 days a week for 4 weeks. IDs in sham r-TMS group only wore the device and did not receive real stimulation therapy. The HCs did not receive this therapy.

### MRI protocol

MRI images were obtained using 3.0 Tesla MR scanner (Achieva 3.0 T, Philips Healthcare, Best, the Netherlands) equipped with an eight-channel phased array head coil. 3D-T1, T2-weighted images and DTI images were acquired. The details of the acquisitions are as following. 3D-T1 sequence parameters: Repeated time (TR) = 6.3 ms; Echo time (TE) = 2.8 ms; Inversion time (TI) = 834 ms; Flip Angle (FA) = 7°; Matrix = 256 × 256; Number of layers = 176; Voxel size = 1.0 × 1.0 × 1.0 mm^3^; Thickness = 1 mm; Field of view (FOV) = 256 × 256 mm^2^. The DTI images were acquired using a single echo plane imaging sequence with 32 diffusion gradient directions (b = 1,000 s/mm2), followed by an additional image without diffusion weighting (b = 0 s/mm2). DTI sequence parameters: TR = 10,000 ms; TE = 92 ms; Matrix = 128 × 128; FOV = 256 × 256 mm^2^; Thickness = 2 mm; Voxel size = 2 mm^3^; Number of layers = 72. To detect other brain lesions, T2-weighted images were also obtained, and the scanning parameters were: TR = 2,643 ms; TE = 80 ms; Acquisition matrix = 352 × 213; FOV = 230 × 201 mm^2^; FA = 90°; Thickness = 5 mm; Number of layers = 26.

In the HCs group, MR images were collected once upon enrollment. In the true rTMS group and the sham rTMS group, MR Images were collected within 3 days before and after the therapy.

### The DTI-ALPS index calculation

**Figure 2:**
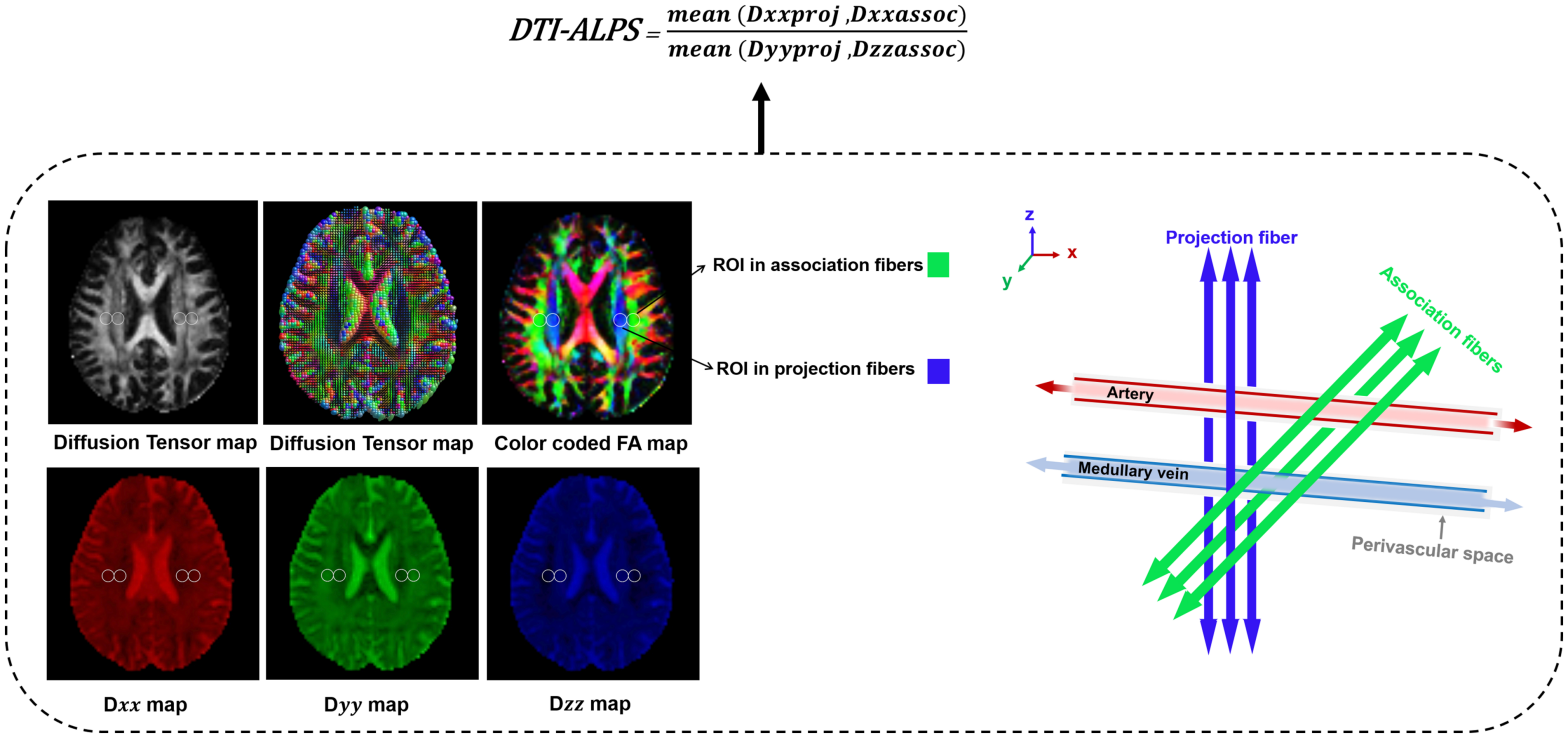
Process illustrating for obtaining DTI-ALPS index.

The DTI-ALPS index was used to evaluate glymphatic function along the perivascular space with use of multidirectional diffusivity maps acquired from DTI data (Bae et al., 2023; Lin et al., 2024; Zhang et al., 2021). The DTI-ALPS index was calculated according with previous study (Zhang et al., 2021). Diffusivities maps were co-registered to the fractional anisotropy (FA) map template^1^ using spm12 package of MATLAB (R2015a). At the top level of the deep vein perpendicular to the lateral ventricle, four 5-mm-diameter regions of interest (ROIs) were set on bilateral projection fibers and association fibers (Figure 2). Then, we manually adjusted the position of the ROIs. The X-axis coordinates of the projection fibers ROIs were located in the center of the blue area, and the association fibers were located in the center of the green area of the color FA map. The x-axis (Dx), y-axis (Dy), and z-axis (Dz) of ROIs on projection and association fibers were recorded as Dxxproj, Dyyproj, Dzzproj, Dxxassoc, Dyyassoc, Dzzassoc, respectively. Then bilateral DTI-ALPS index was calculated as: (Taoka et al., 2017b)


$$\mathrm{DTI}-\text{ALPS index}=\frac{\text{mean}\;(\mathrm{Dx}x\text{proj},\text{Dxxassoc})}{\text{mean}\;(\text{Dyyproj},\text{Dzzassoc})}$$

Two radiologists (G.D. and Z.Z.N. with 9 and 5 years of experience, respectively) blind to clinical data independently placed ROIs and calculated ALPS-index of all subjects for inter-observer reliability analysis.

### Polysomnography

Before and after rTMS therapy, all of IDs underwent standardized PSG by using a portable monitoring system (Philips Alice 6 IDe; Philips Healthcare). Acquisition measures are described in Supplementary material S1. Then, sleep and related events were analyzed according to the rules of the American Academy of Sleep Medicine manual version 2.6. Polysomnographic indices included the followings: total sleep time, sleep efficiency, sleep onset latency, wake after sleep onset, N1 sleep duration, N2 sleep duration, N3 sleep duration, rapid eye movement sleep, arousal index, apnea-hypopnea index, oxygen saturation decreased ≥3% index, mean SpO2 during sleep, maximum obstructive apnea duration, periodic limb movement in sleep index.

### Statistical analysis

Statistical analyses were performed using GraphPad Prism 9.0.0 (GraphPad Software, Inc., La Jolla, CA) and SPSS 27.0 (IBM, Chicago, IL, United States). Continuous variables are expressed by means ± standard deviation (SD) which conform to homoscedasticity and normal distribution. Categorical variables were expressed in frequency. Continuous and categorical variables were compared between three groups using one-way analysis of variance (ANOVA) and ꭓ^2^ tests, respectively. Tukey’s multiple comparisons test was used for further analysis. Interobserver agreement on the DTI-ALPS measurements between the two radiologists was evaluated using the interclass correlation coefficient. Univariate linear regression was used to evaluate the associations between DTI-ALPS and participants’ characteristics, sleep measures and PSG indexes. Non-adjusted and slightly adjusted, and multivariate adjusted models were subsequently conducted using backwards linear regression to identify independent factors of PSG indexes associated with DTI-ALPS. Variables with *p* < 0.2 in the univariate models were included in a backward elimination procedure with *p* removal = 0.1. The paired-samples *t* test was used to assess changes in variables in true/sham rTMS groups before and after therapy. The threshold for statistical significance was *p* < 0.05.

## Results

### Participants characteristics before treatment

This study included 37 IDs [true rTMS group (*n* = 28) and sham rTMS group (*n* = 9)] and 20 HCs. Demographic and cognitive characteristics of the participants are summarized in Table 1. The IDs in both groups had lower MOCA scores before treatment (27.39 ± 2.27 and 25.22 ± 2.68, respectively, *p* < 0.05) compared with HCs (29.45 ± 2.46). In addition, both groups of IDs had higher BAI and BDI scores [BAI (35.78 ± 8.54 and 33.98 ± 5.42), BDI (12.54 ± 7.24 and 17.33 ± 9.41)] before treatment compared to the control group [BAI (26.95 ± 2.51), BDI (4.08 ± 3.71)]. No difference in age, gender, education, or MMSE scores was observed among the three groups (*p* values, 0.17–0.52).

**Table 1:** Demographic and cognitive characteristics of the three groups before rTMS treatment.

Characteristics	HCs (*n* = 20)	True rTMS group (*n* = 28)	Sham rTMS group (*n* = 9)	Statistic	*P*
Age	41.60 ± 9.24	44.39 ± 11.62	50.00 ± 12.18	*F* = 1.83	0.17
Gender				χ^2^ = 3.22	0.20
Female/male	10/10 (50%/50%)	21/7 (75%/25%)	6/3 (66.67%/33.33%)		
Education	14.20 ± 4.29	13.14 ± 4.23	11.22 ± 3.11	*F* = 1.64	0.20
MOCA	29.45 ± 2.46	27.39 ± 2.27^**§**^	25.22 ± 2.68^**§**^	*F* = 5.60	**0.006**
MMSE	28.90 ± 1.77	28.32 ± 1.76	28.44 ± 1.59	*F* = 0.66	0.52
BAI	26.95 ± 2.51	35.78 ± 8.54^**§**^	33.98 ± 5.42^**§**^	*F* = 10.87	**<0.001**
BDI	4.08 ± 3.71	12.54 ± 7.24^**§**^	17.33 ± 9.41^**§**^	*F* = 15.39	**<0.001**

*P* values are for comparison across the three groups before rTMS treatment.

^§^*P* < 0.05 versus the HCs.

Bold values denote statistical significance at the *p* < 0.05 level.

MOCA, Montreal Cognitive Assessment; MMSE, Minimum Mental State Examination; BAI, Beck Anxiety Inventory; BDI, Depression Inventory.

### Comparison of sleep metrics before rTMS therapy among three groups

Sleep profiles of the three groups are summarized in Table 2. Both groups of IDs exhibited significantly poorer sleep performance before treatment, compared to the HCs. The PSQI, ESS and ISI scores were higher in both the group with true rTMS and sham rTMS than in controls before rTMS therapy (*p* < 0.001, *p* = 0.047 and *p* < 0.001, respectively). We observed significant differences in total sleep time, sleep efficiency, sleep onset latency, N2 sleep duration, N3 sleep duration, rapid eye movement sleep and arousal index between the healthy group and the other two groups of IDs. No significant differences in other polysomnographic indices among the three groups (*p* > 0.05).

**Table 2:** Sleep characteristics of the three groups before rTMS treatment.

Characteristics	HCs (*n* = 20)	True rTMS group (*n* = 28)	Sham rTMS group (*n* = 9)	Statistic	*P*
Sleep questionnaires					
PSQI	3.00 ± 1.72	14.71 ± 3.02^**§**^	14.78 ± 3.87^**§**^	*F* = 114.48	**<0.001**
ESS	5.25 ± 3.14	8.04 ± 4.59	8.67 ± 5.02	*F* = 3.23	**0.047**
ISI	1.65 ± 1.27	17.36 ± 3.95^**§**^	14.44 ± 2.46^**§**^	*F* = 161.09	**<0.001**
Polysomnographic measures					
Total sleep time, min	373.70 ± 59.10	286.00 ± 58.50^**§**^	292.80 ± 42.00^**§**^	*F* = 15.06	**<0.001**
Sleep efficiency, %	85.14 ± 7.80	71.13 ± 8.80^**§**^	66.93 ± 6.64^**§**^	*F* = 22.88	**<0.01**
Sleep onset latency, min	16.38 ± 6.74	38.69 ± 15.67^**§**^	41.25 ± 13.23^**§**^	*F* = 20.59	**<0.01**
Wake after sleep onset, min	58.40 ± 41.11	73.55 ± 36.43	84.00 ± 44.02	*F* = 1.56	0.22
N1 sleep duration, min	32.65 ± 12.40	33.79 ± 15.21	36.00 ± 12.92	*F* = 0.180	0.84
N2 sleep duration, min	261.90 ± 32.79	163.50 ± 52.91^**§**^	142.70 ± 30.20^**§**^	*F* = 30.29	**<0.01**
N3 sleep duration, min	53.95 ± 10.60	30.00 ± 13.58^**§**^	27.22 ± 11.44^**§**^	*F* = 25.80	**<0.01**
Rapid eye movement sleep, min	85.65 ± 21.63	41.70 ± 19.38	50.33 ± 15.26	*F* = 28.41	**<0.01**
Arousal index, events/h	5.01 ± 3.96	15.01 ± 7.20^**§**^	16.33 ± 7.04^**§**^	*F* = 18.09	**<0.01**
Apnea-hypopnea index, events/h	1.08 ± 1.56	2.95 ± 5.67	1.40 ± 1.30	*F* = 1.10	0.342
Oxygen saturation decreased ≥3% index, events/h	3.00 ± 4.40	2.37 ± 5.04	1.03 ± 1.08	*F* = 0.56	0.58
Mean SpO_2_ during sleep, %	96.65 ± 1.39	96.51 ± 1.46	97.00 ± 1.12	*F* = 0.42	0.66
Maximum obstructive apnea duration, min	0.09 ± 0.19	0.08 ± 0.17	0.11 ± 0.16	*F* = 0.09	0.91
Periodic limb movement in sleep index, events/h	6.83 ± 12.40	7.43 ± 11.64	7.00 ± 7.12	*F* = 0.02	0.98

*P* values are for comparison across the three groups before rTMS treatment.

^§^*P* < 0.05 versus the HCs.

Bold values denote statistical significance at the *p* < 0.05 level.

PSQI, Pittsburgh Sleep Quality Index; ESS, Epworth Sleepiness Scale; ISI, Insomnia Severity Index.

### Reliability of DTI-ALPS

Interobserver agreement of two radiologists was excellent for the DTI-ALPS index (intraclass correlation coefficient, 0.91 [95% CI: 0.85 to 0.94]) (Supplementary Table S1).

### Comparison of DTI-ALPS before rTMS therapy among three groups

Analysis along the perivascular space index was significantly decreased in true/sham rTMS groups with IDs compared to healthy subjects (Table 3). Dxx values, derived from projection fiber areas, were significantly reduced in true/sham rTMS groups with IDs compared to control subjects (*p* < 0.001). Dxx values derived from association fiber areas, were not significantly reduced between the three groups. Diffusion changes in association fibers and projection fibers show no significant reduction in Dyy and Dzz indices.

**Table 3:** Comparison of the diffusivities and ALPS Indexes among the three groups before rTMS treatment.

	HCs (*n* = 20)	True rTMS group (*n* = 28)	Sham rTMS group (*n* = 9)	Statistic	*P*
Dxxproj	0.64 ± 0.06	0.50 ± 0.04^**§**^	0.57 ± 0.03^**§**^	*F* = 11.30	**<0.001**
Dxxassoc	0.79 ± 0.11	0.77 ± 0.11	0.72 ± 0.09	*F* = 1.04	0.36
Dyyproj	0.46 ± 0.05	0.49 ± 0.07	0.49 ± 0.08	*F* = −1.32	0.28
Dyyassoc	1.01 ± 0.14	1.01 ± 0.15	1.02 ± 0.15	*F* = −1.34	0.27
Dzzproj	1.05 ± 0.06	1.02 ± 0.05	1.05 ± 0.05	*F* = 2.47	0.10
Dzzassoc	0.49 ± 0.10	0.45 ± 0.09	0.44 ± 0.08	*F* = 1.12	0.34
DTI-ALPS	1.67 ± 0.16	1.45 ± 0.17^**§**^	1.39 ± 0.14^**§**^	*F* = 13.08	**<0.001**

*P* values are for comparison across the three groups before rTMS treatment.

^§^*P* < 0.05 versus the HCs.

Bold values denote statistical significance at the *p* < 0.05 level.

Diffusivities are presented as apparent diffusion coefficients (×10–3 mm^2^/s).

Dxassoc: diffusivity along the x-axis in the association fiber area.

Dxproj: diffusivity along the x-axis in the projection fiber area.

Dyassoc: diffusivity along the y-axis in the association fiber area.

Dyproj: diffusivity along the y-axis in the projection fiber area.

Dzassoc: diffusivity along the z-axis in the association fiber area.

Dzproj: diffusivity along the z-axis in the projection fiber area.

### Correlations between sleep metrics of PSG and DTI-ALPS in IDs

Univariate and multivariate regression models were used to evaluate the associations between sleep metrics of PSG and DTI-ALPS in different models. Univariate regression model showed that the DTI-ALPS was negatively correlated with age and arousal index in all IDs (Table 4). DTI-ALPS was positively correlated with total sleep time and N2 sleep duration (Table 4). Other clinical data, cognitive performance scores and sleep questionnaire scores were not correlated with DTI-ALPS (Supplementary Table S2). In the minimally adjusted model I (adjusted for gender and age), the effect size remained significantly positive between DTI-ALPS and N2 sleep duration (β = 0.01, 95%CI: 0.01 to 0.01, *p* = 0.037). In the model II (adjusted for gender, age, education, MOCA, MMSE, BAI, BDI, PSQI, ESS, ISI), the effect size showed a consistent positive association between DTI-ALPS and N2 sleep duration (β = 0.01, 95%CI: 0.01 to 0.01, *p* = 0.034). Similarly, a significant negative correlation between DTI-ALPS and arousal index in both the minimally adjusted model I and rigorously adjusted model II (β = −0.01, 95%CI: −0.02 to −0.01, *p* < 0.001, β = −0.01 and 95%CI: −0.01 to −0.01, *p* = 0.002, respectively).

**Table 4:** Multivariate linear regression of DTI-ALPS with age, total sleep time, N2 sleep duration and arousal index of different models in ID participants before rTMS treatment.

Variables	Crude model		Model I		Model II	
	β (95%CI)	*P*	β (95%CI)	*P*	β (95%CI)	*P*
Age	−0.01 (−0.01 ~ −0.01)	**<0.001**	–	–	–	–
Total sleep time, min	0.01 (0.01 ~ 0.01)	**<0.001**	0.00 (−0.00 ~ 0.00)	0.063	0.00 (−0.00 ~ 0.00)	0.13
N2 sleep duration, min	0.01 (0.01 ~ 0.01)	**0.003**	0.01 (0.01 ~ 0.01)	**0.037**	0.01 (0.01 ~ 0.01)	**0.034**
Arousal index, events/h	−0.02 (−0.02 ~ −0.01)	**<0.001**	−0.01 (−0.02 ~ −0.01)	**<0.001**	−0.01 (−0.01 ~ −0.01)	**0.002**

Bold values denote statistical significance at the *p* < 0.05 level.

CI: Confidence interval.

Model I adjusted for gender and age.

Model II adjusted for gender, age, education, MOCA, MMSE, BAI, BDI, PSQI, ESS, ISI.

### Comparison of true rTMS group and sham rTMS group after rTMS therapy

**Figure 3:**
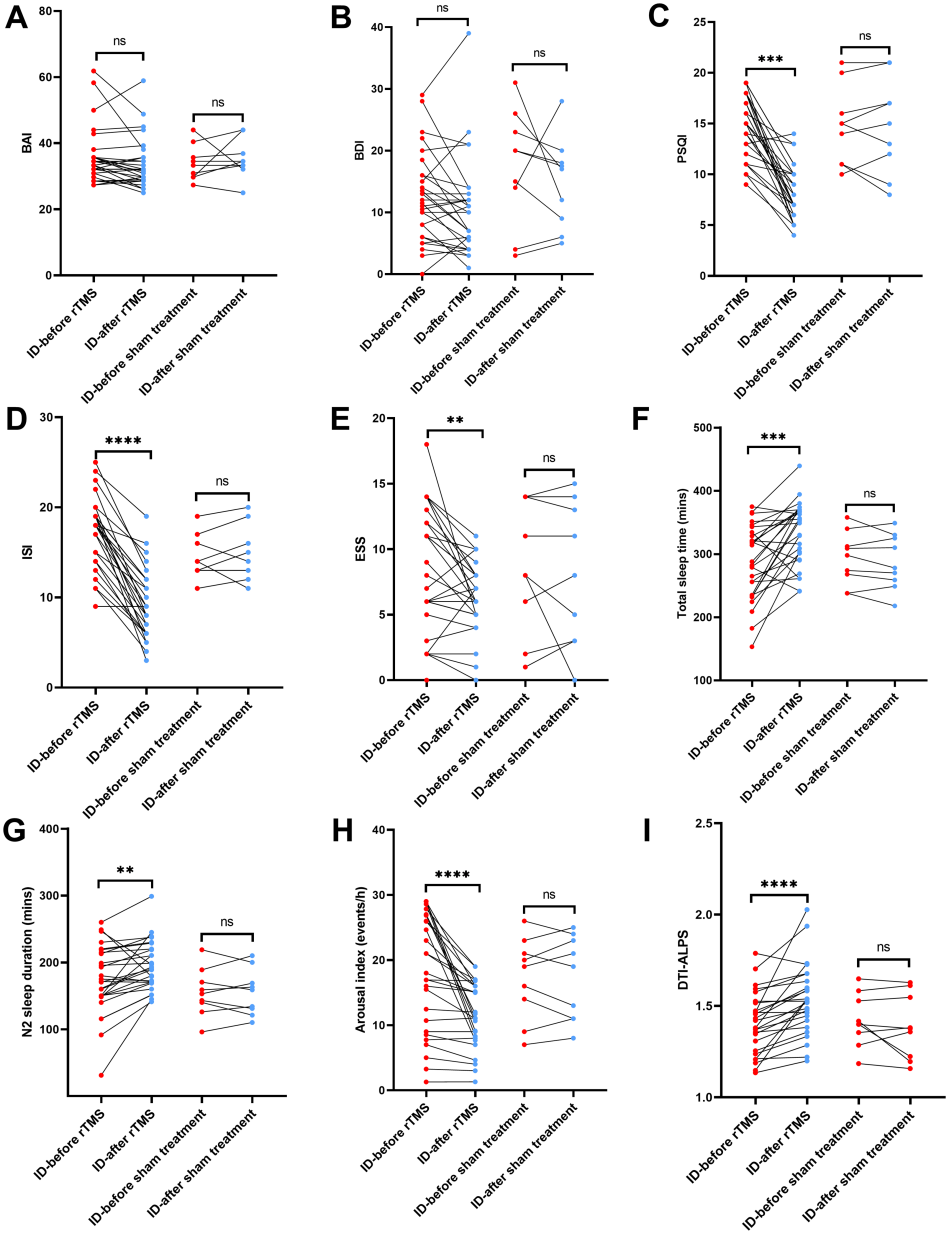
Comparison of metrics in IDs of true and sham rTMS groups before and after rTMS treatment in **(A)** BAI, **(B)** BDI, **(C)** PSQI, **(D)** ISI, **(E)** ESS, **(F)** total sleep time, **(G)** N2 sleep duration, **(H)** arousal index, **(I)** DTI-ALPS.

The true/sham rTMS group with IDs underwent true/sham rTMS therapy, respectively. They were reexamined cognitive performance, sleep questionnaires, sleep metrics of PSG and DTI-ALPS within 3 days after treatment. These participants in the true rTMS group showed a significant decrease in PSQI, ISI, ESS values, arousal index (Figures 3C–E,H) and a significant increase in total sleep time, N2 sleep duration DTI-ALPS index values (Figures 3F,G,I). There was no significant change in the above indexes in the IDs of sham rTMS group (Figures 3A–I).

## Discussion

In this study, we investigated glymphatic function in patients with ID compared with healthy controls by using the DTI-ALPS index and its independent association with sleep parameters of polysomnography, and whether rTMS can improve lymphoid function and sleep status in patients with ID. The main finding of this study was that the DTI-ALPS index was significantly lower in IDs than in healthy controls. Moreover, N2 sleep duration and the arousal index were independently associated with DTI-ALPS index. Our results revealed that rTMS therapy can improve the sleep status and DTI-ALPS index of ID.

In this study, there was no significant difference in gender distribution among the three groups. However, we found that there were more women than men in the true rTMS group and sham rTMS group for ID. This is consistent with previous epidemiological investigations (Berro, 2023). One possible important factor is that ID may be related to hormonal changes specific to women, such as menopause or the late luteal phase of the menstrual cycle. Another consideration is that women are more likely to develop severe depression and anxiety disorders, which are also associated with ID (Krystal, 2003).

At the level of the lateral ventricles, the most direct pathway to the surface of the brain occurs in the radial direction, i.e., the x-axis. The medial artery and vein, as well as their perivascular spaces, run in this x-direction to support the transport outside the brain parenchyma. Here, the diffusion of large nerve fibers dominates in the diffusion image. Therefore, it is necessary to avoid the strong influence of white matter fibers (i.e., projection fibers and association fibers) (Taoka et al., 2017a). The DTI-ALPS index aims to assess the macroscopic movement of water in the perivascular spaces, rather than the movement of water itself. Therefore, it is reasonable to use DTI-ALPS to reflect lymphatic function in this study.

Since DTI-ALPS index was proposed in 2017 (Taoka et al., 2017b), it has been utilized for the assessment the lymphatic system in various studies. Several studies of the DTI-ALPS method in Alzheimer’s disease (Zhang et al., 2024; Matsushita et al., 2024; Chang et al., 2023), Parkinson’s disease (Shen et al., 2022; Meng et al., 2024), small vessel diseases (Tang et al., 2022), idiopathic normal pressure hydrocephalus (Georgiopoulos et al., 2024), traumatic brain injury (Yang et al., 2024), demyelinating disease (Carotenuto et al., 2022), sleep (Saito et al., 2023) and other disease have been reported. However, DTI-ALPS method has several problems. Manual ROI placement can be influenced by subjectivity and arbitrariness. It can only evaluate the effect of the white matter outside the lateral ventricles on the movement of water molecules. The use of a single *b*-value instead of multiple shells makes it impossible to separate the velocity components of diffusion, and the information is considered to be a mixture of diffusion components of different velocities (Taoka et al., 2024). Although the DTI-ALPS index does not reflect the whole of the extremely complex lymphatic system, it may be an indirect indicator of glymphatic system activity. This method is very convenient and non-invasive and can be analyzed retrospectively using a simple formula.

Consistent with our findings, The DTI-ALPS were lower in patients with ID than in healthy controls. The correlation between the glymphatic system and sleep is supported by evidence. Previous findings have suggested that poor sleep quality may disrupt the function of the lymphatic system (Jin et al., 2024), leading to reduced clearance of waste such as Aβ and tau proteins in the brain (Chong et al., 2022; Sangalli and Boggero, 2023). During sleep, the number of glial cells decreases and the interstitial space expands more than in the awake state, thus facilitating the transport of substances within the tissues (Xie et al., 2013). In this study, it was found that the diffusion coefficient value of projection fibers (Dxxproj) was significantly different among the three groups, indicating that the movement of water molecules in the brain parenchyma along the direction of medulla blood vessels was affected in patients with insomnia disorder. Therefore, as reflected in the DTI-ALPS index, there was a significant difference between the three groups.

Sleep divides into nonrapid eye movement (NREM) sleep and rapid eye movement (REM) sleep. Previous research has shown that in mice experiments and in humans, CSF was increased during NREM (Xie et al., 2013; Fultz et al., 2019). The increased flow of CSF during NREM sleep is thought to be related to the flow of the lymphatic system by flushing out CSF to remove toxic proteins. NREM divides into stages N1, N2, and N3, according to the American Academy of Sleep Medicine scoring manual. In this study, N2 sleep duration was independently positive correlation with DTI-ALPS. This is consistent with findings reported by Siow et al. (2022), who observed that the ALPS-index was correlated with N2 sleep duration in 60 years or older adults. N2 sleep, which constitutes the largest percentage of total sleep time in middle-aged adults (Ohayon et al., 2004; Boulos et al., 2019). Previous studies have shown that sleep stages N, especially N2, are predominantly slow-wave activities. Slow-wave activity can lead to a decrease in amyloid in the human brain, which may be related to the active lymphatic system function at this stage (Xie et al., 2013). Therefore, it is easy to understand that ID patients have less N2 and reduced DTI-ALPS.

An independent negative correlation was observed between the arousal index and DTI-ALPS in this study. The increased number of arousal index in ID patients interferes with the normal conversion of sleep cycle and leads to sleep fragmentation, resulting in REM deprivation and sleep structure disorders (Yan et al., 2021), which will inhibit the function of the lymphatic system and reduce the efficiency of metabolic waste removal (Holth et al., 2019). These results suggest recurrent arousal from sleep are responsible for lymphatic dysfunction.

In the present study, we also found that rTMS therapy can improve the sleep status and DTI-ALPS index of ID. This is consistent with previous study. Two systematic reviews that summarizing studies on rTMS in patients with ID found that the rTMS improve sleep architecture monitored by PSG (Krone et al., 2023; Lanza et al., 2023). There may be molecular biological mechanisms by which rTMS therapy can improve lymphatic function. On the one hand, rTMS enhances the drainage efficiency of the lymphatic system and meningeal lymphatic vessels, and reduces the activation of microglia and astrocytes (Lin et al., 2021). On the other hand, cerebral arterial pulsation is an important driver of paravascular CSF-interstitial fluid exchange (Gaberel et al., 2014), and that changes in arterial pulsation may contribute to the clearance of toxic solute in the brain (Iliff et al., 2013). After rTMS treatment, cerebral cortical activity was increased, which was accompanied by changes in vascular hemodynamics (Allen et al., 2007; Liu et al., 2023). Therefore, this may also be a potential mechanism by which rTMS affects the intracranial lymphatic system.

This study has several limitations. First, the sample size was relatively small, and the study was conducted at a single center. Second, there is no detailed study on the classification of insomnia disorder patients. Third, this study was limited to short-term effects of rTMS. There are no follow-up data on the efficacy of rTMS therapy in the medium and long term. Follow-up of persistent changes in lymphoid function and sleep indicators will improve the clinical applicability of this study. Forth, rTMS adopted the traditional 5 cm localization method, and the therapeutic target might be slightly different.

## Conclusion

Our findings indicate that DTI-ALPS index is significantly lower among IDs, due to glymphatic system dysfunction. We also found independent associations of the DTI-ALPS index with N2 sleep duration and the arousal index. The rTMS can be used as a treatment for IDs.

## Data Availability

The raw data supporting the conclusions of this article will be made available by the authors, without undue reservation.

## Footnotes

^1^ https://neurovault.org/images/1406/
