# Supplementary material for: The change of MRI indexes of brain glymphatic function and sleep status before and after repeated transcranial magnetic stimulation in insomnia disorder patients
Source: Front Neurosci. 2025 May 30;19:1545885. doi: 10.3389/fnins.2025.1545885 (PMC12162663; doi:10.3389/fnins.2025.1545885)
Supplement: Supplementary file 1 [file Data_Sheet_1.doc]

**The change of MRI indexes of brain glymphatic function and sleep status before and after repeated transcranial magnetic stimulation in insomnia disorder patients**

**Supplementary Material**

**Content**

[Supplementary Material S1 2](#__RefHeading___Toc194484070)

[Polysomnography Protocol 2](#__RefHeading___Toc194484071)

[Supplementary Material Table S1 3](#__RefHeading___Toc194484072)

[Interobserver agreement of two radiologists on diffusivities and the DTI-ALPS. 3](#__RefHeading___Toc194484073)

[Supplementary Material Table S2 4](#__RefHeading___Toc194484074)

[Univariate Linear Regression of DTI-ALPS with clinical data, cognitive performance scores and sleep questionnaire scores in ID participants before rTMS treatment. 4](#__RefHeading___Toc194484075)

# Supplementary Material S1

# Polysomnography Protocol

All subjects were required to sleep in the ward for 2 consecutive nights, adapt to the environment in the first night, and start formal records in the second night. The subjects fell asleep individually in a controlled room temperature ranging from 18 to 26℃. They were responsible for setting their own sleep and wake times. The experimental sleep conditions were consistent with the formal testing conditions, and any data that could not accurately reflect the subjects' usual sleep patterns due to environmental changes during the night would be excluded from analysis. Menstrual cycles were not taken examination.

The application uses the Philips Alice 6 Ide polysomnography. The electrodes are silver-plated with a diameter of 1 cm and have a total of 4 leads. Two of the leads are for the electro-oculogram placed 1 cm outside and downward (or upward) from the outer corner of the left (or right) eye, with the reference electrode placed on the earlobe. One lead is for the electromyography, placed 1.5 cm lateral to the midline of the lower jaw. The other lead is for the electroencephalogram (C3-A2). The signals are input into a multi-channel physiological recording instrument, amplified, and recorded. The time constant is 0.3 seconds, the high-frequency filter is set to 30 Hz, the gain is 1, and the calibration voltage is 50 mV.

# Supplementary Material Table S1

# Interobserver agreement of two radiologists on diffusivities and the DTI-ALPS.

| Value | Reader 1 | Reader 2 | ICC | 95% CI |
| --- | --- | --- | --- | --- |
| DTI-ALPS | 1.52 ± 0.19 | 1.50 ± 0.22 | 0.91 | 0.85-0.94 |
| Dxxproj (×10^−3^ mm^2^/sec) | 0.60 ± 0.056 | 0.60 ± 0.073 | 0.733 | 0.59-0.83 |
| Dxxassoc (×10^−3^ mm^2^/sec) | 0.77 ± 0.10 | 0.76 ± 0.12 | 0.95 | 0.92-0.97 |
| Dyyproj (×10^−3^ mm^2^/sec) | 0.47±0.066 | 0.48±0.079 | 0.92 | 0.87-0.95 |
| Dyyassoc (×10^−3^ mm^2^/sec) | 1.02±0.15 | 1.02±0.16 | 0.83 | 0.81-0.96 |
| Dzzproj (×10^−3^ mm^2^/sec) | 1.03±0.054 | 1.03±0.067 | 0.79 | 0.66-0.87 |
| Dzzassoc (×10^−3^ mm^2^/sec) | 0.46±0.094 | 0.47±0.096 | 0.85 | 0.82-0.97 |

ICC: intraclass correlation coefficient

ICC ≥0.75, excellent agreement;

ICC 0.60–0.74, good agreement;

ICC 0.40–0.59, fair agreement;

ICC < 0.40, poor agreement.

Dxassoc: diffusivity along the x-axis in the association fiber area,

Dxproj: diffusivity along the x-axis in the projection fiber area,

Dyassoc: diffusivity along the y-axis in the association fiber area,

Dyproj: diffusivity along the y-axis in the projection fiber area,

Dzassoc: diffusivity along the z-axis in the association fiber area,

Dzproj: diffusivity along the z-axis in the projection fiber area,

ALPS: analysis along the perivascular space

# Supplementary Material Table S2

# Univariate Linear Regression of DTI-ALPS with clinical data, cognitive performance scores and sleep questionnaire scores in ID participants before rTMS treatment.

| Variables | *P* | β (95%CI) |
| --- | --- | --- |
|
| Age | **<.001** | -0.01 (-0.01 ~ -0.01) |
| Education | 0.911 | 0.00 (-0.01 ~ 0.01) |
| MOCA | 0.196 | 0.01 (-0.01 ~ 0.03) |
| MMSE | 0.632 | -0.01 (-0.04 ~ 0.02) |
| BAI | **0.029** | -0.01 (-0.01 ~ -0.01) |
| BDI | 0.482 | -0.00 (-0.01 ~ 0.00) |
| PSQI | 0.430 | -0.01 (-0.03 ~ 0.01) |
| ESS | 0.667 | -0.00 (-0.01 ~ 0.01) |
| ISI | 0.450 | -0.01 (-0.02 ~ 0.01) |
| Total sleep time, min | **<.001** | 0.01 (0.01 ~ 0.01) |
| Sleep efficiency, % | 0.707 | -0.00 (-0.01 ~ 0.01) |
| Sleep onset latency, min | 0.723 | 0.00 (-0.00 ~ 0.00) |
| Wake after sleep onset, min | 0.959 | 0.00 (-0.00 ~ 0.00) |
| N1 sleep duration, min | 0.797 | -0.00 (-0.00 ~ 0.00) |
| N2 sleep duration, min | **0.003** | 0.01 (0.01 ~ 0.01) |
| N3 sleep duration, min | 0.400 | 0.00 (-0.00 ~ 0.01) |
| Rapid eye movement sleep, min | 0.665 | 0.00 (-0.00 ~ 0.00) |
| Arousal index, events/h | **<.001** | -0.02 (-0.02 ~ -0.01) |
| Apnea-hypopnea index, events/h | 0.875 | -0.00 (-0.01 ~ 0.01) |
| Oxygen saturation decreased ≥3% index, events/h | 0.219 | -0.01 (-0.03 ~ 0.01) |
| Mean SpO_2_ during sleep, % | 0.134 | -0.03 (-0.07 ~ 0.01) |
| Maximum obstructive apnea duration, min | 0.525 | -0.11 (-0.45 ~ 0.23) |
| Periodic limb movement in sleep index, events/h | 0.942 | -0.00 (-0.01 ~ 0.01) |

Bold values denote statistical significance at the *p* < 0.05 level.

CI: Confidence Interval; BDI: Beck Depression Inventory; BAI Beck Anxiety Inventory; MOCA: Montreal Cognitive Assessment; MMSE: Minimum Mental State Examination; PSQI: Pittsburgh Sleep Quality Index; ESS: Epworth Sleepiness Scale; ISI: Insomnia Severity Index.
